# Beta and Angiotensin Blockades Are Associated With Improved 10‐Year Survival in Renal Transplant Recipients

**DOI:** 10.1161/JAHA.112.000091

**Published:** 2013-02-22

**Authors:** Waqas Aftab, Padmini Varadarajan, Shuja Rasool, Arputharaj Kore, Ramdas G. Pai

**Affiliations:** 1 Loma Linda University, Loma Linda, CA (W.A., P.V., S.R., A.K., R.G.P.)

**Keywords:** ACE inhibitor, β blocker, kidney transplant, survival

## Abstract

**Background:**

Mortality in allograft kidney transplant recipients is high, and cardiovascular disease is the leading cause of death in these patients. They have heightened activity of sympathetic and renin–angiotensin systems. We tested the hypothesis that blockade of sympathetic and renin–angiotensin systems in these patients may offer a survival benefit using a large cohort of patients with long‐term follow up.

**Methods and Results:**

Medical records of 321 consecutive patients from our institution who had received renal transplantation between 1995 and 2003 were abstracted. Survival was analyzed as a function of pharmacological therapies adjusted for age, sex, and comorbidities. The characteristics of the 321 patients were as follows: age at transplant, 44±13 years; 40% male; 89% with hypertension; 36% with diabetes, and mean left ventricular ejection fraction of 60%. Over a follow‐up of 10±4 years, there were 119 deaths. Adjusted for age, sex, diabetes, and coronary artery disease, use of a beta‐blocker therapy (*P*=0.04) and angiotensin‐converting enzyme inhibitor or receptor blocker (*P*=0.03) was associated with better survival. This treatment effect was seen across all major clinical subgroups and was supported by propensity score analysis. The propensity score–adjusted 10‐year survival was 95% in those taking both groups of medications, 72% in those taking either of them, and 64% in those taking neither (*P*=0.004).

**Conclusions:**

Use of beta‐blocker and angiotensin blocking therapies is associated with higher survival after renal transplantation, indicating their potential protective role in this high‐risk population.

## Introduction

Cardiovascular disease remains the leading cause of death in kidney transplant recipients.^1^ Although the use of antihypertensive agents in the posttransplant period has consistently shown improved graft survival, their role in long‐term patient survival remains uncertain because of the lack of prospective trial data.^2^ The National Kidney Foundation does not favor one antihypertensive agent over others in the treatment of posttransplant hypertension.^3^ However, because of graft safety and feasibility of their use with cyclosporine‐based immunosuppression regimens, calcium channel blockers (CCBs) are generally used as first‐line agents. There is some reluctance to use beta‐blockers (BBs) or angiotensin‐converting enzyme inhibitors or angiotensin receptor blockers (ABs) because of fear of graft hypoperfusion, posttransplant hyperkalemia, and the development of new‐onset diabetes mellitus.^4^

Both renal failure and posttransplant patients have increased activity of sympathetic nervous and renin–angiotensin systems, a situation similar to heart failure syndrome.^5–11^ Hence, we evaluated the effect of BB and AB therapies, alone or in combination, on long‐term survival in this high‐risk population with high cardiovascular mortality.

## Methods

### Study Population

The study was approved by our Institutional Review Board. We had a total of 321 patients who had received any form of kidney transplant at our institute between January 1995 and December 2003. Chart reviews were performed by a medical resident and a cardiology fellow, and data on demographics, comorbidities, and pharmacological variables were collected. In patients with echocardiograms, the left ventricular ejection fraction was assessed by a level 3 trained echocardiographer.

### Definition of Comorbidities

Hypertension was defined as blood pressure >140/90 mm Hg or a history of hypertension and being on antihypertensive medications. Diabetes mellitus was defined as fasting blood sugar >125 mg/dL or being on a regimen of antidiabetic medication. Coronary artery disease (CAD) was deemed present if any of the following were present: a history of angina pectoris, myocardial infarction, a positive stress test, angiographic evidence of CAD, coronary intervention, coronary artery bypass surgery, or presence of significant wall motion abnormalities on the echocardiogram. In the absence of angiographic data on all patients, it is possible that the prevalence of CAD could be underestimated. Major adverse cardiac events (MACEs) were defined as sudden cardiac death, fatal and nonfatal myocardial infarction (MI), new arrhythmias or ECG changes requiring coronary care unit stay or direct current cardioversion therapy, pulmonary edema, and new‐onset congestive heart failure. Non‐ST‐elevation MI was defined as typical rise and fall of cardiac enzymes (troponin or Ck‐MB) in the setting of chest pain with or without ST‐T depression.

### Pharmacological Data

Pharmacological data were collected as a single‐point posttransplant use of aspirin, BB, calcium channel blocker (CCB), and AB. The therapies were broadly categorized, and details of different agents or their doses were not collected.

### Mortality Data

Patients were censored during the last week of July 2010, and deaths were confirmed using the secured Social Security Index Web site.

### Statistical Analysis

Analysis was performed using Stat View 5.01 (SAS Institute Inc, Cary, NC). Characteristics of patients with and without BB and AB were compared using the Student *t* test for continuous variables and the chi‐square test for categorical variables. Statistical tools used for survival analysis included the Kaplan–Meier method, Cox regression model, and propensity score analysis. Propensity score analysis was used in an attempt to adjust for group differences between treated and untreated groups. Probability of receiving a BB (propensity score) for each patient was modeled by using logistic regression conditioned on the covariate values for that individual including age, sex, coronary disease, diabetes, hypertension, AB therapy, and duration of dialysis. Effect of BBs on survival was analyzed adjusting for this propensity score using the Cox regression model. In a similar fashion, propensity score analysis was performed to analyze the effect of ABs on survival as well. *P*≤0.05 was considered significant. As described later, propensity score analysis was used as well.

## Results

### Patient Characteristics

Patient characteristics are shown in Table 1. The mean age of the recipients was 44±13 years (range, 15 to 78 years) at the time of transplant, 60% were male, there was diabetes mellitus in 36%, hypertension in 89%, dyslipidemia in 23%, and coronary artery disease in 20%, the left ventricular ejection fraction was 60±16%. A total of 77% of patients who received a transplant had been on dialysis for 1 to 5 years, 18% for 6 to 10 years, and 3% for >10 years. A total of 86 patients were on a BB, 98 on an AB, 181 on a CCB, and 32 on aspirin.

**Table 1.** Patient Characteristics

Variable	
Total number of patients	321
Age, y	44±13
Male	60%
Smoking	13%
Diabetes mellitus	36%
Hypertension	89%
Hyperlipidemia	23%
Diabetes and hypertension	35%
Hemodialysis duration, y	
1 to 5	77%
5 to 10	18%
>10	3%
Chest pain	9%
NYHA symptom class	
I	1%
II	91%
III	5%
IV	0
LVEF	60±16%
EF	
≥40%	89%
<40%	11%
Any coronary artery disease	18%
Prior coronary revascularization	6%
Aspirin use	10%
BB use	27%
AB use	31%
CCB use	56%
BB and AB	11%
BB and CCB	18%
AB and CCB	18%

NYHA indicates New York Heart Association; LVEF, left ventricular ejection fraction; BB, β‐blocker; AB, angiotensin blocker; CCB, calcium channel blocker.

### Univariate Predictors of Survival

Over a period of 10±4 years, there were 119 deaths. As shown on Table 2, the univariate predictors of higher mortality included age at transplant >45 years (HR, 2.66; 95% CI, 1.84 to 3.85; *P*<0.0001), diabetes mellitus (HR, 2.12; 95% CI, 1.47 to 3.00; *P*<0.0001), prior myocardial infarction (HR, 2.6; 95% CI, 1.46 to 4.78; *P*=0.001), and MACE following transplant (HR, 2.9; 95% CI, 1.7 to 5.1; *P*=0.002). Treatment with a BB (HR, 0.58; 95% CI, 0.36 to 0.92; *P*=0.02) or AB therapy (HR, 0.58; 95% CI, 0.37 to 0.90; *P*=0.01) was associated with lower mortality. Sex, smoking, hypertension, hyperlipidemia, left ventricular ejection fraction, duration of dialysis, and use of a CCB or aspirin had no association with survival.

**Table 2.** Univariate Correlates of Survival

Variable	HR	95% CI of HR	*P* Value
Age >45 y	2.66	1.84 to 3.85	<0.0001
Female sex	0.92	0.64 to 1.33	0.67
Smoking	1.39	0.84 to 2.30	0.19
Hypertension	1.07	0.60 to 1.90	0.81
Diabetes mellitus	2.12	1.47 to 3.05	<0.0001
Dyslipidemia	1.28	0.84 to 1.94	0.23
Prior MI	3.15	1.76 to 5.62	0.0001
MACE	2.95	1.68 to 5.16	0.0002
Any CAD	1.60	0.95 to 2.71	0.08
BB use	0.58	0.36 to 0.92	0.02
AB use	0.58	0.37 to 0.90	0.01

HR indicates hazard ratio; CI, confidence interval; MI, myocardial infarction; MACE, major adverse cardiac event; CAD, coronary artery disease; BB, β‐blocking agent; AB, angiotensin‐blocking agent.

### BB Therapy and Survival

**Figure 1.**
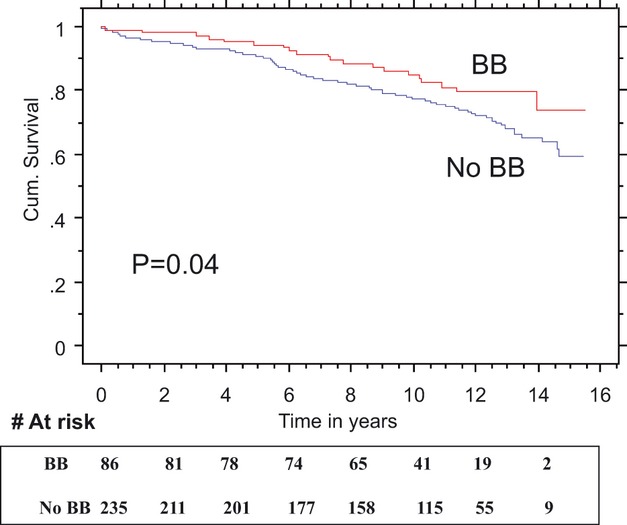
Survival curves of patients with and without β‐blocker (BB) therapy adjusted for propensity score.

In the 86 patients on BB therapy, the 10‐year survival was higher compared with those not on a BB adjusted for the propensity score (HR, 0.61; CI, 0.37 to 0.98; *P*=0.04; Figure 1). The protective effect of BBs was seen in patients with both lower and upper halves based on propensity scores for BB use and was consistent across clinical subgroups based on the presence or absence of hypertension, diabetes mellitus, myocardial infarction, and perioperative adverse cardiac events (Table 3). It is noteworthy that the benefit of a BB was seen in those without prior myocardial infarction or left ventricular systolic dysfunction. Adjusted for group differences, as shown in Table 4, using the Cox regression model, use of a BB was associated with better survival (*P*=0.04).

**Table 3.** β‐Blocker Subgroup Analysis

Subgroups	HR	95% CI	*P* Value
Patients with diabetes mellitus	0.36	0.14 to 0.90	0.03
Patients without diabetes mellitus	0.74	0.41 to 1.33	0.32
Patients with hypertension	0.57	0.36 to 0.92	0.02
Patients with hyperlipidemia	0.93	0.38 to 2.27	0.89
Patients with normal lipids	0.47	0.26 to 0.83	0.009
Patients with prior myocardial infarction	1.36	0.30 to 6.1	0.70
Patients with no prior myocardial infarction	0.57	0.35 to 0.94	0.02
Patients with major cardiac adverse events	0.30	0.08 to 1.13	0.07
Patients with no major cardiac adverse events	0.59	0.36 to 0.97	0.04

HR indicates hazard ratio; CI, confidence interval.

**Table 4.** Correlates of BB Therapy

Variable	On BB (n=86)	Not on BB (n=235)	*P* Value
Age, y	42±12	45±13	0.07
Female sex	40%	41%	0.71
Hypertension	99%	85%	0.0005
Diabetes mellitus	25%	40.8%	0.010
Hyperlipidemia	20%	24%	0.5
Smoking	9%	14%	0.2
Left ventricular ejection fraction	61±13	60±12	0.5
Ejection fraction <40%	9%	7%	0.6
Major adverse cardiac events	8%	6%	0.4
Prior myocardial infarctions	4%	7%	0.2
Dialysis <5 y	66%	80%	0.007
Concomitant aspirin use	11%	10%	0.3
Concomitant AB use	40%	27%	0.03
Concomitant CCB use	68%	52%	0.009

BB indicates β‐blocker; AB, angiotensin blocker; CCB, calcium channel blocker.

### AB Therapy and Survival

**Figure 2.**
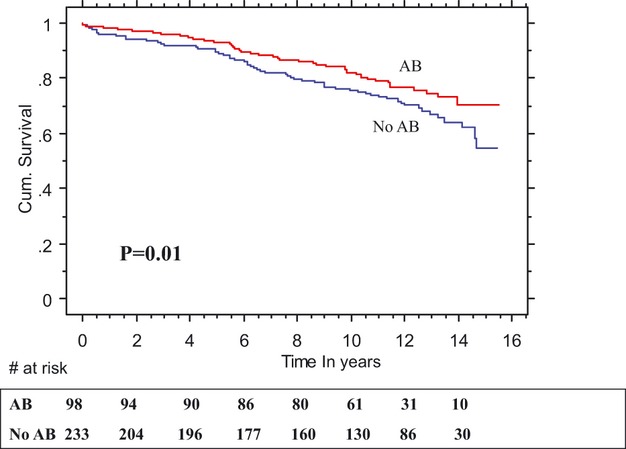
Survival curves, adjusted for propensity score, of patients with and without angiotensin‐blocking (AB) therapy with an angiotensin receptor blocker or an angiotensin‐converting enzyme inhibitor.

In the 98 patients on AB therapy, the 10‐year survival was higher compared with those not on an AB adjusted for the propensity score (HR, 0.54; CI, 0.34 to 0.86; *P*=0.01; Figure 2). The protective effect of AB was seen in patients with both lower and upper halves based on propensity scores for AB use and was consistent across clinical subgroups based on the presence or absence of hypertension, diabetes mellitus, myocardial infarction, and perioperative adverse cardiac events (Table 5). It is noteworthy that the benefit of AB was seen in those without diabetes mellitus or left ventricular systolic dysfunction. As shown in Table 6, adjusted for group differences using the Cox regression model, use of AB was associated with better survival (*P*=0.03).

**Table 5.** AB Subgroup Analysis

Subgroup	HR	95% CI	*P* Value
Patients with hypertension	0.59	0.38 to 0.92	0.02
Patients with diabetes mellitus	0.67	0.38 to 1.18	0.17
Patients without diabetes mellitus	0.37	0.17 to 0.81	0.01
Patients with hyperlipidemia	0.37	0.16 to 0.88	0.02
Patients with normal lipids	0.67	0.40 to 1.14	0.14
Patients with prior myocardial infarction	0.17	0.02 to 1.34	0.09
Patients with no prior myocardial infarction	0.65	0.41 to 1.025	0.06
Patients with major adverse cardiac events	1.14	0.35 to 3.70	0.82
Patients with no major adverse cardiac events	0.54	0.34 to 0.88	0.01

AB indicates angiotensin blocker; HR, hazard ratio; CI, confidence interval.

**Table 6.** Correlates of AB therapy

Variable	On AB (n=98)	Not on AB (n=223)	*P* Value
Age, y	42±13	45±13	0.08
Female sex	24%	24%	0.50
Hypertension	98%	85%	0.0005
Diabetes mellitus	45%	33%	0.05
Hyperlipidemia	32%	19%	0.010
Smoking	13%	11%	0.46
LVEF	58±14	61±11	0.16
EF <40%	17%	4%	0.003
MACE	6%	7%	0.8
Prior myocardial infarction	5%	7%	0.6
Dialysis <5 y	79%	76%	0.57
Concomitant aspirin use	9%	10%	0.8
Concomitant CCB use	59%	55%	0.53
Concomitant BB use	35%	23%	0.03

AB indicates angiotensin blocker; LVEF, left ventricular ejection fraction; MACE, major adverse cardiac event; CCB, calcium channel blocker; BB, β‐blocker.

### Combined BB and AB Therapy and Survival

**Figure 3.**
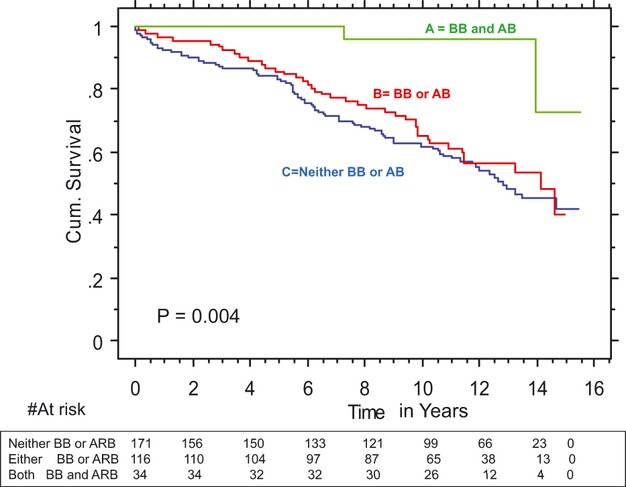
Survival curves, adjusted for propensity score, of patients who took β‐blocker (BB) and angiotensin‐blocking (AB) therapy vs those who took neither or either. ARB indicates angiotensin receptor blocker.

As therapies with BBs and ABs were both associated with better survival, we analyzed the survival patterns of patients who were taking either both or none of these medications adjusted for propensity scores for their use. As shown in Figure 3, the adjusted 10‐year survival was 95% (95% CI, 87% to 100%) in those taking both groups of medications, 72% (95% CI, 63% to 81%) in those taking either of them, and 64% (95% CI, 57% to 71%) in those taking neither, suggesting an additive benefit (*P*=0.004).

### Mutivariable Cox Proportional Hazard Ratio Analysis

All univariate predictors of survival with a *P*≤0.10 were entered into a Cox regression model. As shown in Table 7, greater age (*P*<0.0001), diabetes mellitus (*P*=0.0002), prior myocardial infarction (*P*=0.006), major perioperative cardiac event (*P*=0.001), and lack of BB therapy (*P*=0.04) or AB therapy (*P*=0.03) were found to be independent predictors of higher mortality.

**Table 7.** Multivariable Predictors of Survival

Variable	Multivariable HR	95% CI	*P* Value
Age >45 y	2.39	1.62 to 3.53	<0.0001
Diabetes mellitus	1.99	1.38 to 2.89	0.0002
Prior MI	2.28	1.26 to 4.13	0.006
MACE	2.65	1.46 to 4.83	0.001
BB use	0.60	0.36 to 0.98	0.04
AB use	0.61	0.38 to 0.96	0.03

HR indicates hazard ratio; CI, confidence interval; MI, myocardial infarction; MACE, major adverse cardiac event; BB, β‐blocking agent; AB, angiotensin‐blocking agent.

## Discussion

Our study provides important insights into survival after renal transplant and possible protective role offered by BB and AB therapies. Although there are conflicting data on the use of AB on long‐term survival after kidney transplant, there is no information about BB use. In general, common apprehensions about BB use are worsening of diabetes mellitus and decrease of cardiac output and renal hypoperfusion.

### BB Therapy After Renal Transplantation

Our study is the first to show the mortality benefit of BB therapy in renal transplant recipients. In these patients, BBs are primarily prescribed for control of hypertension. Their role as cardioprotective medications is less appreciated here, even in high‐risk groups with prior myocardial infarctions and heart failure. Because of the perceived risk of decreased cardiac output, resulting in decreased renal perfusion, altered glomerular filtration with nonselective beta‐blockers such as propranolol,^12^ increased proteinuria with cardioselective BBs such as atenolol,^13^ serious hyperkalemia with labetolol,^14^ and development of new onset DM^15^ or masking of symptoms of hypoglycemia, BBs are not overwhelmingly prescribed. They have been shown to reduce mortality by 23% in diabetic patients in a post‐MI setting and all‐cause mortality by 16% in those with congestive heart failure.^16^ There is growing clinical evidence that supports the possible cardioprotective role of BBs in patients with end‐stage renal disease (ESRD) with or without diabetes. Foley et al^17^ in the USRDS Wave 3 and 4 Studies noted clear survival benefit of use of beta blockers as antihypertensive in dialysis patients. In an observational study of hemodialysis patients without previously documented heart failure, Abbott et al^18^ were able to show reduced risk of new heart failure, cardiovascular‐related death, or any‐cause mortality with the use of beta‐blockers. Wali et al^19^ in their meta‐analysis of randomized trials of patients with moderate renal disease and heart failure and Cice et al^20^ in their randomized placebo‐controlled study of ESRD patients with cardiomyopathy over a period of 2 years have demonstrated survival benefit from the use of carvedilol. In our study, the survival benefit of BBs was observed across the cohort and was not just limited to hypertensive patients. The benefit was consistent in all subgroups, including those without a prior myocardial infarction or heart failure. The possible mechanism by which BBs may offer such protection across the whole cohort and not just in high‐risk or hypertensive patients could be their effect on reducing sympathetic nervous system activity level. This activity is markedly increased in ESRD patients because of signals generated by the failing kidneys that are sent to the hypothalamus via afferent nerve fibers^21^ and decreased production of renalase,^22^ a mono amine oxidase that plays a pivotal role in catecholamine metabolism. The net effect is high plasma catecholamine levels that are associated with increased cardiovascular events and mortality in hemodialysis patients.^5^ In posttransplant patients, high sympathetic activity continues even after renal transplantation and resolution of uremia, unless denervation and removal of native kidneys are performed.^6^ This activity further increases in the first few weeks after surgery, especially in patients who are on cyclosporine‐based immunosuppressive regimens.^7^ This is the most vulnerable period, when the postoperative MACE rate and mortality are high. Thus, the use of BBs in these few weeks may be most beneficial and may afford the most cardioprotection. Another possible mechanism by which BBs may work in the long term is reduction of proinflammatory cytokines, which are high in ESRD patients and contribute significantly to the creation of atherosclerotic plaque.^8^

Among the common reasons associated with decreased prescription of BBs in posttransplant patients, perhaps the most serious is the perceived reduction in renal perfusion and increased vascular resistance, especially in patients on cyclosporine A based immunosuppressant regimens. This perceived risk has been tested and refuted. Branten et al,^23^ in a study of 12 renal transplant recipients on cyclosporine A treatment, showed that beta‐blockers do not significantly alter renal perfusion or other measures of renal hemodynamics in these patients. In nontransplant patients, beta‐blockers have actually been shown to reduce vascular resistance and improve renovascular hemodynamics.^24^ Furthermore, there is ample scientific evidence to suggest that the negative metabolic impact of these agents such as aberrations of glucose or lipid metabolism that may also play a role in overall decreased use of these agents is mostly seen with B1 selective or nonselective blockers and can be ameliorated by the addition of alpha 1 blockage.^25–27^

Our study is large and examines post–renal transplant patients over the long term. In addition, it shows that BBs may prolong survival of these patients, even when a traditional prophylactic indication for BBs does not exist.

### AB and Survival

Another interesting observation in our study is the potential benefit of AB just like BBs on long‐term survival in posttransplants patients. The effect was independent of and additive to BB use and was observed throughout the cohort and not just in hypertensive or high‐risk patients. As is the case with BBs, there is some degree of apprehension about the use of AB in posttransplant patients when the donor is of advanced age and when there is prolonged cold ischemia, fear of hyperkalemia, induction of anemia in the recipient, or decreased renal blood flow.^28^ The renal protective effects of AB in hypertensive patients with proteinuria after kidney transplant have been described in a number of studies.^9–10,13^ The safety data on AB use in posttransplant period are very promising,^11^ and there is an increasing trend of AB prescription, from <20% in the early 1990s to >45% in the 2000s.^28–29^ The role of these agents in patient survival remains a matter of debate. Although Tutone et al^30^ in their longitudinal follow‐up of 634 posttransplant patients and Heinze et al^31^ in their retrospective study of >2000 posttransplant patients have shown clear benefit of AB for patient survival, Opelz et al^29^ in their analysis of a cohort of >17 000 patients have strongly argued against it. To date, the only prospective trial designed to have addressed mortality after renal transplant was the SECRET trial with candesartan, which was terminated prematurely because of low event rates, although candesartan did improve blood pressure and proteinuria significantly compared with the placebo arm.^32^ In our study, the overall rate of AB prescription was 30%. The majority of the patients who were prescribed AB were hypertensive (98%). These results are promising and argue for the more frequent use of these agents in posttransplant population. The biological, clinical, and pharmacological effects of AB go beyond their traditional antihypertensive properties, elevation of bradykinin levels and blocking angiotensin II at angiotensin type I receptors. Clinical trial evidence indicates improved patient survival across the spectrum of systolic heart failure in post‐MI setting and severe symptomatic congestive heart failure, as well as in asymptomatic patients treated with AB.^33–35^ Further, there are strong clues about reduced vascular events in patients with normal LV function treated with these agents irrespective of the blood pressure reduction.^36^ There are several possible mechanisms by which these medications may offer survival benefit in ESRD and posttransplant patients. First, just like BBs, but probably not to the same extent, ABs also reduce sympathetic tone,^37^ which, as mentioned above, has been shown to be related to increased mortality of these patients. Second, renin–angiotensin system activation in peripheral blood vessels has been shown to increase the production of endogenous vasoconstrictor endothelin I via angiotensin I and angiotensin II receptor–mediated activation of nuclear transcription factor‐kb.^38–39^ In kidney transplant recipients treated with cyclosporine, nitric oxide levels are reduced and endothelium‐dependent vasodilatation is impaired.^40^ Amore et al^41^ in their animal study showed that cyclosporine‐mediated vasoconstriction can be prevented by administration of l‐arginine (increasing nitric oxide). With the inhibition of the renin–angiotensin system, the production of endothelin I is modulated, and by simultaneously increasing local levels of bradykinin, which leads to increased levels of nitric oxide levels, AB actually may shift the vascular balance from local vasoconstrictors to vasodilators, promoting a healthier endothelial environment and providing cardioprotection.

### Study Limitations

The main limitation of our study is its retrospective, observational nature. We have attempted to adjust for covariates using the Cox regression model and propensity score analysis. The latter is reported to eliminate up to 85% of bias associated with observational studies.^42–43^ Unfortunately, there are no prospective randomized studies adequately addressing these questions. The data on medication use were 1 time documented in posttransplant follow‐up. Details on duration and intensity of therapy were difficult and not possible to measure. In view of the large number of medications in each group, we did not collect data on individual medications in BB or AB groups. Another major limitation of our study is the lack of allograft function and graft loss data as patients went back to their primary nephrologists. We also lacked data on race and details of immunosuppressive therapy.

### Summary and Conclusions

Beta‐blocker and angiotensin‐blocking therapies were associated with better long‐term survival in 321 renal transplant recipients. This treatment effect was seen across all major clinical subgroups and was supported by propensity score analysis. Propensity score–adjusted 10‐year survival was 95% in those taking both groups of medications, 72% in those taking either of them, and 64% in those taking neither, indicating their potential protective role in this high‐risk population (*P*=0.004). We conclude that these therapies are safe and potentially beneficial in renal transplant patients and recommend a randomized trial to confirm these observational findings.
